# Reactive Oxygen Species Mediated Prostaglandin E_2_ Contributes to Acute Response of Epithelial Injury

**DOI:** 10.1155/2017/4123854

**Published:** 2017-02-09

**Authors:** Yi-Ping Hu, Yin-Bo Peng, Yi-Fan Zhang, Ying Wang, Wei-Rong Yu, Min Yao, Xiu-Jun Fu

**Affiliations:** ^1^Department of Burns and Plastic Surgery, No. 9 People's Hospital, and Institute of Traumatic Medicine, Shanghai Jiao Tong University School of Medicine, Shanghai 201900, China; ^2^Department of Burns and Plastic Surgery, Guangzhou Red Cross Hospital, Jinan University, Guangzhou 510220, China

## Abstract

Reactive oxygen species (ROS) generated after tissue injury play a crucial role during wound healing through initiating acute inflammation, clarifying infection and dead tissue, and mediating various intracellular signal transduction. Prostaglandin E_2_ (PGE_2_) has been identified as one of the major factors responsible for inflammation and tissue repair. In this study, we tested our hypothesis that ROS produced by damaged human keratinocytes induces the synthesis of PGE_2_. In vitro epithelial wounding model was used to observe the production of ROS and secretion of PGE_2_ as well as the involved signal pathway. The mechanical injury caused the rapid production of ROS in in vitro cultured keratinocytes, which was significantly blocked by an inhibitor of nicotinamide adenine dinucleotide phosphate oxidase. The increased intracellular ROS caused by mechanical injury stimulates PGE_2_ production in a time-dependent manner via the activation of cyclooxygenase-2 (COX-2), which was stimulated by phosphorylation of extracellular signal-regulated protein kinase (ERK). These results indicate ROS-induced ERK activation leading to the activation of COX-2 and the synthesis of PGE_2_ in human keratinocytes responding to mechanical injury in the acute phase.

## 1. Introduction

Cutaneous wound healing is a complex yet well-organized process, which includes three interactive phases of inflammation, proliferation, and tissue reconstruction. Injury, as the signal for initiating wound healing, triggers the process of tissue repair by activating the directly or indirectly wound-involved cells, for example, epithelial cells, endothelial cells, fibroblasts, and inflammatory cells. The traditional concept widely accepts that the chemotaxis of leukocytes to the wound site successively follows the activation of hemostasis after injury, and reactive oxygen species (ROS) are mainly produced by leukocytes through “respiratory burst” for disinfection and debridement. However, a recent study revealed that a fast and dramatic increase of ROS at the wound margin was essential for rapid recruitment of leukocytes to the wound site [1]. ROS produced by injured epithelial cells through the activation of nicotinamide adenine dinucleotide phosphate (NADPH) oxidase (NOX) play a critical role in initiating inflammatory response of wound healing. Additional studies also show that, in contrast to oxidative damage at high concentration, ROS serve as intracellular and extracellular signaling messengers and regulate numerous downstream signal transduction and gene expression at low concentration [2, 3]. ROS have been determined to be able to activate mitogen-activated protein kinases (MAPKs) cascades [4–7]. Comprised of extracellular signal-regulated protein kinase (ERK), c-Jun N-terminal kinase (JNK), and p38 kinase, MAPKs are the core molecules of cell stress-response signaling network. MAPKs are protein kinases specific to the amino acids serine, threonine, and tyrosine, which are involved in directing the cellular response to a diverse array of stimuli including mechanical damage. Activated by the injury signals, MAPKs coordinate cell functions including proliferation, differentiation, migration, survival or apoptosis, and gene expression during wound healing [6, 8, 9].

Cyclooxygenase (COX) enzyme and its enzymatic product prostaglandin E_2_ (PGE_2_) are known to be critical inflammatory factors in the early phase of wound healing [10]. Among the two isoforms of COX, COX-1 and COX-2, COX-1 is expressed constitutively in most tissues and may be responsible for housekeeping functions. In contrast, COX-2 is not detectable in most normal tissues, but its expression can be induced by endotoxin, cytokines, growth factors, and carcinogens [11, 12]. PGE_2_ is believed to be strongly associated with the signs of redness, swelling, heat, and pain in the wound area, which indicates its role as a pivotal proinflammatory factor. It has been confirmed that PGE_2_ has a profound influence on wound repair by affecting proliferation and migration of epithelial cells, vascular tone, regional blood flow, vascular permeability and remodeling, and angiogenesis [13, 14].

As ROS have been demonstrated to activate many intracellular molecules and upregulate oxidative stress-related genes during wound healing, we hypothesized that ROS might modulate the production of PGE_2_ through MAPKs pathway on mechanical damaged human keratinocytes, and the increased PGE_2_ might then be responsible for cell injury and repair and inflammatory cell recruitment. In the present study, we sought to investigate the effects of ROS on PGE_2_ production in human keratinocytes after mechanical injury, as well as the possible mechanisms.

## 2. Materials and Methods

### 2.1. Cell Culture and Treatment

The immortalized human skin keratinocyte cell line, HaCat, from the Institute of Basic Medical Sciences, Chinese Academy of Medical Sciences, was grown in RPMI-1640 medium (Gibco, BRC) supplemented with 10% FBS at 37°C with 5% CO_2_. Cells were seeded at 6-well plates or 60 mm dishes for further experiments. After 48 hr of incubation, cells grew to 70%–80% confluence, and the medium was replaced with RPMI-1640 containing 1% FBS for overnight before the indicated treatment. The undamaged cells worked as control. When assessing the effects of ROS inhibitor, ERK inhibitor, or COX-2 inhibitor, cells were pretreated with diphenyleneiodonium (DPI) (Sigma, MO, USA) for 30 min, PD98059 (Beyotime, Shanghai, China) for 1 hr, or NS398 (Beyotime, Shanghai, China) for 1 hr, respectively, before injury.

### 2.2. In Vitro Wounding Model

Freshly isolated HaCat cells growing to 70%–80% confluence in 6-well plate or 60 mm dish were used for wounding model. Briefly, cells were mechanically removed from the plate or dish by dragging a 20 *μ*L pipette tip linearly on the confluent monolayer as the plate rested over a template. The spacing between the scratching lines was 3 mm.

### 2.3. ROS Detection

Cells grown in 60 mm culture dishes were incubated for 15 min at 37°C in dark with 10 *μ*M of carboxy-H_2_DCFDA (Invitrogen, CA, USA). The cells were then washed three times with Phosphate-Buffer Saline (PBS) and exposed with scratching injury for indicated time at 37°C. When assessing the effect of ROS inhibitor, cells were pretreated with 10 *μ*M DPI for 30 min. The cells were harvested and centrifuged for 5 min at 10,000 rpm at 4°C to remove the supernatants. After the pellets were resuspended in PBS, levels of ROS were detected using a fluorospectrophotometer (Molecular Devices, USA) with 480 nm excitation and 530 nm emission.

### 2.4. Western Blot Analysis

Cells were washed with PBS, harvested using a scraper, and solubilized in cold cell lysis buffer (Beyotime, Shanghai, China). Aliquots of lysate were heated for 5 min at 95°C. Equal amounts of lysate were subjected to SDS-polyacrylamide gel electrophoresis on 10% gels and were transferred onto a PVDF membrane (Millipore, USA). The membranes were blocked with 5% nonfat dry milk in 0.01 M Tris-buffered saline (PH 7.4) containing 0.05% Tween-20 (TBST) at room temperature for 1 hr. The membranes were then incubated with primary antibodies of ERK, phosphorylated ERK (p-ERK) rabbit monoclonal antibody (mAb), or COX-2 rabbit mAb (1 : 1000) (Cell Signaling Technology, MA, USA) overnight at 4°C after they were incubated with appropriate HRP-conjugated secondary antibodies. The protein bands on the blots were detected with enhanced chemiluminescence detection kit (Thermo Scientific, IL, USA) according to the manufacturer's instructions.

### 2.5. COX Activity Assessment

More than 1 × 10^9^ cells in culture were harvested using a scraper and resuspended in 100 *μ*L of a cell lysis buffer containing 1 mM EDTA. The lysed cells were centrifuged at 2000 rpm at 4°C for 10 min, and the supernatant was discarded. Cell pellets were resuspended in 400 *μ*L of a lysis buffer containing 0.1 M Tris-HCl, 1 mM EDTA, homogenized with ice ultrasonic for 30 s, and then centrifuged at 10000 rpm at 4°C for 15 min. The supernatant was assayed with the COX fluorescence activity assay kit (Cayman Chemical CO, MI, USA), following the manufacturer's instructions.

### 2.6. Measurement of PGE_2_ Release

Supernatants of HaCat culture were collected and concentrated by centrifugation. The concentration of PGE_2_ was determined by PGE_2_ ELISA Kit (Cayman Chemical Co., MI, USA), according to the manufacturer's instructions.

### 2.7. Statistical Analysis

Results were expressed as means ± SEMs. Statistical analysis was performed using Student's *t*-test and ANOVA. Those *p* values that were less than 0.05 were considered statistically significant.

## 3. Results

### 3.1. Scratching Injury Induces Generation of ROS

**Figure 1:**
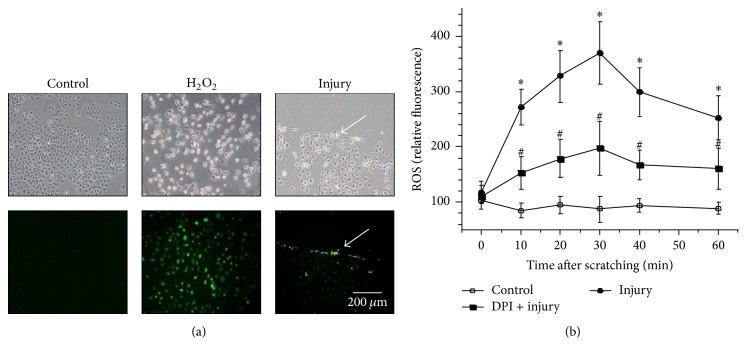
Rapid formation of ROS induced by scratching injury in HaCat cells. (a) The intracellular ROS were visualized by the reaction with probe carboxy-H_2_DCFDA under fluorescent microscopy. Cells without damage worked as negative control, while H_2_O_2_ treated cells were used as positive control. (b) The quantities of ROS were shown after scratching for undamaged control cells, damaged cells, and damaged cells plus DPI treatment. Data are representative of three independent experiments with triplicate samples. *∗* and # indicate *p* < 0.05 compared with undamaged control cells and with scratched cells, respectively.

In order to understand the effects of ROS on epithelial wound healing, the levels of intracellular ROS in HaCat cells were measured after injury. HaCat cells were injured by manual scratches, and the assessments of ROS levels were made using fluorescence probe carboxy-H_2_DCFDA, which can be oxidized to fluorescent fluorescein by ROS in cells (Figure 1(a), center column). As shown in Figure 1, scratching injury caused a rapid increase in intracellular ROS (Figure 1(a), right column) in a time-dependent manner with a maximal response within 30 min (3.5-fold) and sustained to 60 min (3-fold) (Figure 1(b)). In addition, pretreatment with a NOX inhibitor, DPI, for 30 min dramatically blocked the scratching-induced production of ROS (1.8-fold after 30 min and 1.5-fold after 60 min) in HaCat cells (Figure 1(b)). These data suggest that mechanical injury can induce rapid production of ROS during wound healing.

### 3.2. Scratching Injury Increases Synthesis of PGE_2_ via ROS

**Figure 2:**
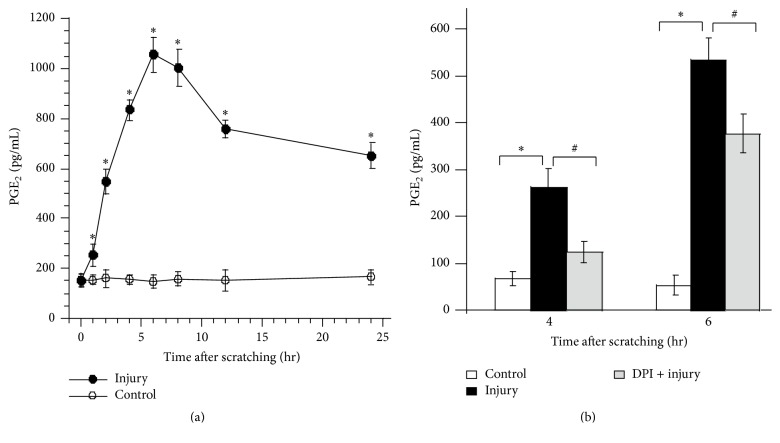
Scratching-injury-induced PGE_2_ production and the despondence on ROS. (a) PGE_2_ secretion by HaCat cells after scratches. PGE_2_ was measured by ELISA in different periods of time as indicated. (b) Suppressed secretion PGE_2_ by NOX/ROS inhibitor. Values are means ± SEMs of three replicates. *∗* and # indicate *p* < 0.05 compared with undamaged control cells and with scratched cells, respectively.

Recent evidence has shown that ROS have multiple downstream targets and play a key role in triggering production of proinflammatory factors, such as PGE_2_. Thus, to determine whether there is change of the level of PGE_2_ after epithelial damage, we investigated the release of PGE_2_ after scratching injury on HaCat cells in vitro. The level of PGE_2_ was significantly increased at 6 hr after injury and remained greater than the undamaged control for at least 24 hr in HaCat after injury (Figure 2(a)).

In order to know the relationship between the generation of ROS and the secretion of PGE_2_ after scratching injury, the inhibitor of NOX, DPI, was used to pretreat the cells, and then the levels of PGE_2_ were measured. The level of PGE_2_ in the DPI treatment plus scratching injury cells was significantly lower than that in the injury only cells at 4 hr or 6 hr after injury, respectively (Figure 2(b)). Our data indicate that scratching injury-induced PGE_2_ synthesis is mediated by ROS.

### 3.3. ERK Activation Is Involving in Scratching-Injury-Induced Enhancements of ROS and PGE_2_

**Figure 3:**
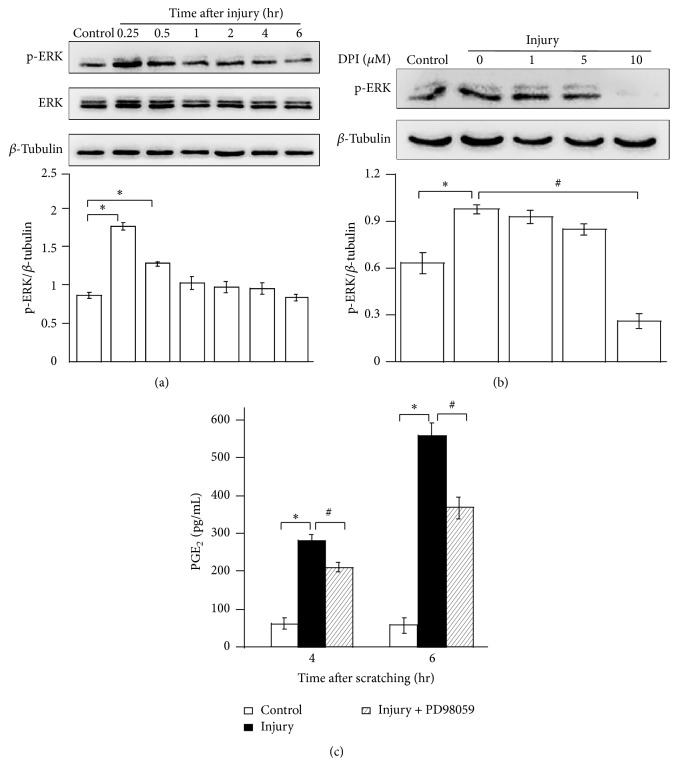
Participation of ERK activation in scratching injury-induced release of ROS and PGE_2_. (a) The change of p-ERK and ERK in HaCat cells treated with scratching injury for different periods of time as indicated. The protein levels of p-ERK and ERK were determined by immunoblot analysis and normalized to *β*-tubulin. (b) The level of p-ERK was decreased in HaCat cells preincubated with different concentrations of DPI for 30 min followed by scratches for 0.5 hr. (c) The dependence of PGE_2_ production caused by scratching injury on ERK activation. Values are means ± SEMs of three replicates. *∗* and # indicate *p* < 0.05 compared with undamaged control cells and with scratched cells, respectively.

ROS have also been reported to play a major role as second messengers and to contribute to the activation of signaling pathway. In an effort to understand the mechanism underlying the injury-induced generation of ROS and synthesis of PGE_2_, ERK activation after scratching injury was investigated. As illustrated in Figure 3(a), scratching injury increased the extent of ERK phosphorylation, which peaked at 15 min and returned to basal levels after 60 min. No significant change in total ERKs was found after injury. These data suggest that scratching injury can activate ERK signaling in HaCat cells.

To examine whether ROS generation is involved in injury-stimulated activation of ERK, we tested the effect of DPI (NOX/ROS inhibitor) on ERK phosphorylation after injury. HaCat cells were or were not pretreated with DPI for 30 min before scratches, and then phosphorylated ERK was determined using Western blotting.  Figure 3(b) showed that 1 *μ*M or 5 *μ*M DPI pretreatment did not significantly attenuate injury-induced ERK activation 15 min after injury. However, 10 *μ*M DPI pretreatment almost completely blocked injury-induced phosphorylation of ERK. Taken together, the data indicated that ROS are critical mediators of the injury-induced activation of ERK in HaCat cells.

Further, the effect of ERK activation on the injury-induced release of PGE_2_ was investigated. Incubation of HaCat cells with ERK inhibitor, PD98059, reduced injury-induced PGE_2_ production (Figure 3(c)). This suggested that ERK activation is located upstream of injury-induced PGE_2_ release in HaCat cells. However, it should be noted that the ERK is only partially responsible for PGE_2_ release induced by injury, since ERK inhibitor did not completely block PGE_2_ release.

### 3.4. Injury-Induced Synthesis of PGE_2_ Is Dependent on COX-2, Not COX-1

**Figure 4:**
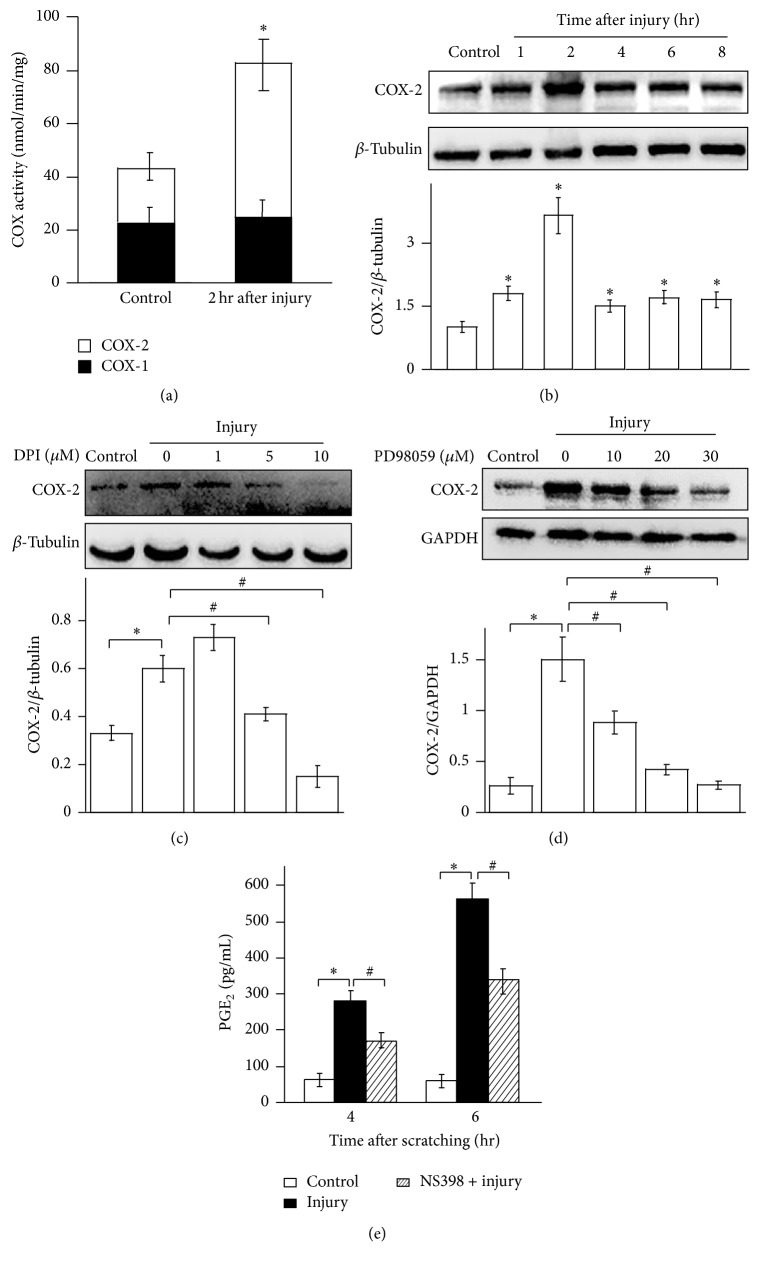
Injury-induced PGE_2_ release mediated by COX-2, not COX-1. (a) COX-1 and COX-2 activity of uninjured or injured HaCat cells were measured by COX activity assay. (b) COX-2 expression was increased in HaCat cells treated with scratching injury for different periods of time as indicated. The protein levels of COX-2 were determined by immunoblot analysis and normalized to *β*-tubulin. (c) The induced expression of COX-2 in HaCat cells was suppressed by NOX/ROS inhibitor DPI. (d) The induced expression of COX-2 in HaCat cells was suppressed by ERK inhibitor. (e) The induced expression of COX-2 in HaCat cells was suppressed by a COX-2 inhibitor. Values are means ± SEMs of three replicates. *∗* and # indicate *p* < 0.05 compared with undamaged control cells and with scratched cells, respectively.

Several reports have illustrated that COX, the major rate-limiting enzyme, involved in the synthesis of PGE_2_. For understanding the activity COX after injury, we tested its activity by COX activity assay. As illustrated in Figure 4(a), injury significantly upgraded COX-2 activity at 2 hr after scratching. However, low level of COX-1 was detected after injury and had no difference compared to the level of undamaged control cells. After that, COX-2 protein expression levels were evaluated by Western blotting. Enhanced expression of COX-2 was examined just 1 hr after scratching in HaCat cells, with a continuous enhancement 2–8 hr after injury (Figure 4(b)). These data suggested that COX-2, not COX-1, is dramatically induced by injury. To corroborate the link between injury-induced ROS production, ERK phosphorylation, and COX-2 generation, we used DPI or PD98059 to treat HaCat cells before injury. Figures 4(c) and 4(d) showed that incubation of HaCat cells with the different dose of DPI or PD98059 decreased injury-induced COX-2 generation. Our data illuminated that ROS production and ERK activation are responsible for COX-2 induction. To further confirm a role of COX-2 for injury-induced PGE_2_ synthesis, NS398, a specific COX-2 inhibitor, was used to determine PGE_2_ production after injury in HaCat cells. A decrease in injury-induced PGE_2_ induction by the addition of NS398 was detected via ELISA (Figure 4(e)). In summary, the stimulation of PGE_2_ was mediated by the activation of COX-2 but not COX-1 after injury. It should be noted, however, that COX-2 is not the only molecule responsible for PGE_2_ secretion induced by injury as COX-2 inhibitor did not fully block PGE_2_ release.

## 4. Discussion

In this study, we investigated the molecular mechanisms by which ROS stimulated the production of proinflammatory mediator PGE_2_ in human keratinocytes with an in vitro injury model. We found that the increased intracellular ROS caused by mechanical injury stimulates PGE_2_ production in a time-dependent manner via the activation of COX-2 in human keratinocytes. Additionally, this stimulation requires activation of the signal pathway through phosphorylation of ERK, which contributes to COX-2 induction and PGE_2_ synthesis. These results suggest that ROS are not only potent oxidant involved in cellular injury response, but also second messengers activating signal pathway and modulating inflammatory mediators. To our knowledge, this is the first demonstration of ROS-induced ERK phosphorylation leading to the activation of COX-2 and the synthesis of PGE_2_ in mechanically injured human keratinocytes.

Cutaneous wound healing commences with blood coagulation followed by infiltration of neutrophils and macrophages to the wound site, which then release a large amount of ROS for anti-infection and debridement. Deficiency of ROS production in phagocytes is responsible for the chronic granulomatous disease, which can cause impaired wound healing in human [15]. Nevertheless, the excess of ROS including superoxide anion (O_2_^−^), hydrogen peroxide (H_2_O_2_), and hydroxyl radical (^∙^OH) is primarily regarded as damage to cells during tissue regeneration. In contrast to a large amount of ROS produced in phagocytes, low level of ROS secreted in a sustained manner has been reported in multiple wound healing cells including keratinocytes [16], fibroblasts [17], and endothelial cells [18]. These low concentrations of ROS participate in a variety of biological processes, including epithelialization, angiogenesis, and granulomatous tissue formation [2, 4]. This study indicates that mechanical injury induces rapid production of ROS in human keratinocyte, which is one of the major cells responsible for wound healing. And the rapidly increased ROS in injured keratinocyte are mainly produced by NOX, as the specific inhibitor of this enzyme significantly blocked the scratching-induced production of ROS. This result is consistent with previous in vivo observation of enriched H_2_O_2_ production in the wound site of mouse dermal wound model [2].

Using a full-thickness incisional model of normal wound in mice, the previous study has shown that, during the acute phase of wound healing, PGE_2_ is the predominant proinflammatory mediate derived from metabolite [10]. PGE_2_ may play a key role in initiating the early inflammation and recruiting cells of the immune system to the site of injury. The increased level of PGE_2_ after wounding was confirmed in the in vitro cultured human keratinocytes induced by scratching injury. In addition, the production of PGE_2_ after injury in keratinocytes was significantly attenuated by NOX/ROS inhibitor DPI. Similar findings have been demonstrated in UVA radiated keratinocytes [19] and H_2_O_2_ treated endothelial cells [13]. COX-2 was established as the key enzyme involved in the production of PGE_2_ under abnormal conditions. COX-2 rather than COX-1 was found, in this study, to be responsible for the increased PGE_2_ in injured keratinocytes based on the evidence of dramatically enhanced expression of COX-2 protein. These indicate that ROS-COX2/PGE_2_ is one of the critical intracellular signal pathways accounting for acute response to mechanical injury in keratinocytes. Multiple prostanoid and leukotriene receptors have been identified on keratinocytes [14], indicating that PGE_2_ produced by keratinocytes can influence the proliferation, migration, and reepithelialization during wound healing in a manner of autocrine. In addition, PGE_2_ secreted by keratinocytes could influence the process of wound healing by modulating the proliferation of fibroblast [20] and affecting the vascular tone, regional blood flow, and angiogenesis [13] through paracrine.

A very common targeted molecule by ROS inside cell is protein tyrosine phosphorylation (PTP), which controls the phosphorylation of tremendous proteins involving cellular signal transduction. MAPKs, as well as phosphoinositide 3-kinase (PI3K), have been identified to be involved ROS-mediated intracellular activities [4]. The activation of ERK (one of the MAPKs) by ROS is related to cell propagation and migration, which contributes to wound healing [8, 9] and the invasion and metastasis of malignant tumors [11]. Increased extent of ERK phosphorylation was identified in scratching injured HaCat, while the total ERKs did not increase significantly. ERK phosphorylation was further demonstrated to be suppressed by NOX/ROS inhibitor DPI dramatically, which confirms that NOX/ROS is indispensable for damage-induced activation of ERK in HaCat. ERK phosphorylation was reported in macrophage treated with oxidized low density lipoprotein, and p-ERK further upregulated COX-2 mRNA and protein expression [21, 22]. In the current study, COX-2 rather than COX-1 was confirmed to be activated p-ERK in HaCat. The important inflammatory mediator PGE_2_ production in HaCat was declined by ERK inhibitor, PD98059, which suggests the upstream mediation of ERK on the PGE_2_ release after mechanical injury.

In summary, the NOX/ROS-p-ERK-COX-2/PGE_2_ pathway is proved by the present study to be involved in the reaction of human keratinocytes to acute mechanical injury. Our results shed a light on the mechanisms accounting for cellular signal transduction in the acute phase of epithelial wound injury, which may be beneficial for developing future therapeutic approaches for tissue repair.
